# Strand-Specific Reverse Transcription PCR for Detection of Replicating SARS-CoV-2

**DOI:** 10.3201/eid2702.204168

**Published:** 2021-02

**Authors:** Catherine A. Hogan, ChunHong Huang, Malaya K. Sahoo, Hannah Wang, Becky Jiang, Mamdouh Sibai, Marisa Holubar, Roshni Mathew, James Zehnder, Benjamin A. Pinsky

**Affiliations:** Stanford University School of Medicine, Stanford, California, USA (C.A. Hogan, C. Huang, M.K. Sahoo, H. Wang, M. Holubar, R. Mathew, J. Zehnder, B.A. Pinsky);; Stanford Health Care, Stanford (C.A. Hogan, B. Jiang, M. Sibai, B.A. Pinsky)

**Keywords:** SARS-CoV-2, RNA, infection control, testing, COVID-19, respiratory infections, severe acute respiratory syndrome coronavirus 2, 2019 novel coronavirus disease, coronavirus disease, zoonoses, viruses, coronavirus, diagnosis, screening, minus strand, PCR

## Abstract

We developed an assay that detects minus-strand RNA as a surrogate for actively replicating severe acute respiratory syndrome coronavirus 2. We detected minus-strand RNA in 41 persons with coronavirus disease up to 30 days after symptom onset. This assay might inform clinical decision-making about patient infectiousness.

Real-time reverse transcription PCR (rRT-PCR) is the standard diagnostic method for coronavirus disease 2019, but it cannot differentiate between actively replicating and inactive virus. Active replication is a critical factor for infectiousness; however, its time course is difficult to estimate because of the typical 20–50 days before rRT-PCR negative conversion occurs (*1*,*2*). PCR cycle threshold (C_t_) values might help physicians to determine a patient’s infectiousness, but researchers have isolated replicating virus from patients with a wide range (28–33) of C_t_ values (*3*–*7*). Given the stringent biosafety precautions needed for viral culturing of severe acute respiratory syndrome coronavirus 2 (SARS-CoV-2), physicians require additional diagnostic tools. Actively replicating virus produces minus-strand RNA intermediates that can be detected by PCR (*8*,*9*). We developed and validated a 2-step strand-specific rRT-PCR for the detection of actively replicating SARS-CoV-2 and assessed its clinical performance.

## The Study

We conducted standard nucleic acid amplification testing at the Stanford Health Care Clinical Virology Laboratory (Stanford, CA, USA) using the Panther Fusion SARS-CoV-2 Assay (Hologic Inc., https://www.hologic.com), the Panther Aptima SARS-CoV-2 Assay (Hologic Inc.), or the in-house rRT-PCR specific to the SARS-CoV-2 envelope gene (permitted by Emergency Use Authorization) (*10*,*11*). We did not culture SARS-CoV-2 because we did not have access to a biosafety level 3 laboratory.

We developed a novel 2-step rRT-PCR specific to the minus strand of the envelope gene (Appendix). First, we used strand-specific primers to convert SARS-CoV-2 RNA to complementary DNA. Then, we amplified the complementary DNA by rRT-PCR in 3 separate positive, negative, and background (no primer) reactions using the Rotor-Gene Q instrument (QIAGEN, https://www.qiagen.com) (Appendix). We conducted the analytical validation during May–June 2020. We used in vitro transcribed minus- and plus-strand RNA to evaluate the linearity, precision, and lower limit of detection of the assay (Appendix).

We retrospectively collected a convenience set of upper respiratory specimens with a broad range of C_t_ values. These samples had been collected and frozen from 93 inpatients and outpatients who were treated at Stanford Health Care and tested positive for SARS-CoV-2 during March 12–April 9, 2020. We also reviewed the electronic medical records of the participating patients. For the prospective phase of the study, we collected upper respiratory samples from 53 consecutive patients with confirmed SARS-CoV-2 infection by standard rRT-PCR during July 31–September 4, 2020 (Appendix). Treating physicians ordered strand-specific rRT-PCR on the basis of clinical need; we used samples from these patients in the prospective phase.

We conducted analytical validation (*12*) and statistical analysis using Stata version 15.1 (StataCorp LLC., https://www.stata.com) (Appendix). We considered a 2-tailed p<0.05 to be significant. This study was approved by the Stanford Institutional Review Board (protocol no. 48973).

**Figure 1:**
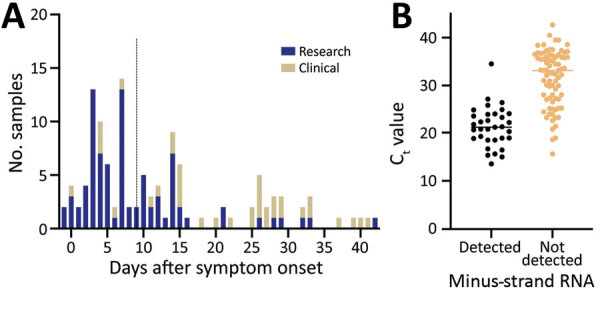
Frequency distribution of days between symptom onset and testing in study on strand-specific real-time reverse transcription PCR for detection of replicating severe acute respiratory syndrome coronavirus 2, California, USA, 2020. Dashed line indicates the median number of days since symptom onset. B) Distribution of standard real-time reverse transcription PCR cycle threshold values by results of strand-specific real-time reverse transcription PCR. Horizontal line indicates median.

**Figure 2:**
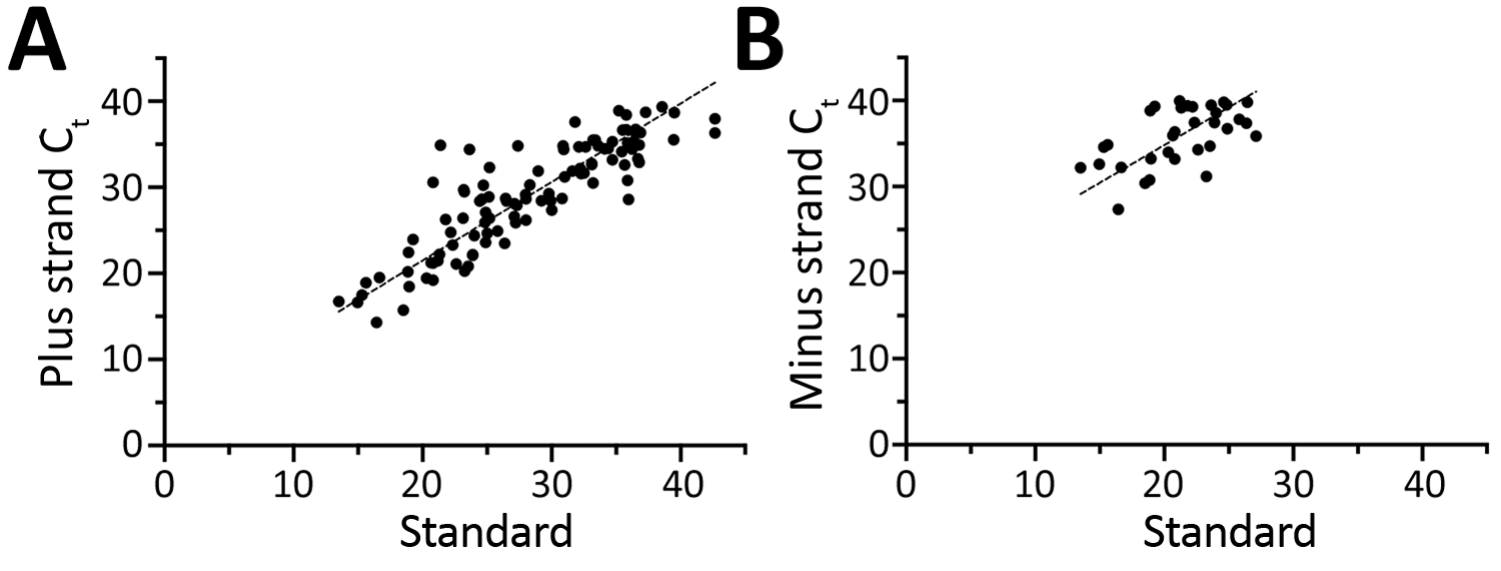
Deming regression analysis of C_t_ values by strand-specific real-time reverse transcription PCR as a function of the C_t_ values by standard real-time reverse transcription PCR for severe acute respiratory syndrome coronavirus 2. Results of PCR for plus strand (A; y = 0.91x + 3.26) and minus strand (B; y = 0.88x + 17.30). C_t_, cycle threshold.

In total, we analyzed specimens from 146 patients: 93 in the retrospective phase and 53 in the prospective phase (Appendix Tables 3, 4). The median age was 50 years (interquartile range 36–63 years); 73 (50.0%) were women, 26 (17.8%) were immunocompromised, and 30 (20.5%) were admitted to the intensive care unit for coronavirus disease during the course of the study (Table 1). Samples were collected a median of 9 days (interquartile range 4–18 days) after symptom onset (Figure 1, panel A). We detected minus-strand RNA in 41 (28.1%) patients. The median C_t_ value of samples with detected minus-strand RNA (20.7) was significantly lower than those in which the minus strand was not detected (33.2; p<0.01) (Figure 1, panel B). The results of this strand-specific assay were closely correlated with the standard rRT-PCR results (Figure 2, panels A, B). The ratio of minus:plus strands varied by patient within 14 days after symptom onset (Appendix Figure 2). 

**Table 1:** Characteristics of patients in study on strand-specific PCR for SARS-CoV-2, California, USA, 2020*

Characteristic	Total	Minus strand detected		Unadjusted p value†	Adjusted p value‡
		Yes	No		
Total	146 (100.0)	41 (100.0)	105 (100.0)		
Median age, y (IQR)	50 (36–63)	61 (42–73)	48 (35–58)	<0.01	<0.01
Sex					
F	73 (50.0)	21 (51.2)	52 (49.5)	0.9	0.9
M	73 (50.0)	20 (48.8)	53 (50.5)		
Initial treatment location					
Emergency room	100 (68.5)	29 (70.7)	71 (67.6)	0.8	0.9
Outpatient	37 (25.3)	9 (22.0)	28 (26.7)		
Inpatient	9 (6.2)	3 (7.3)	6 (5.7)		
Concurrent conditions					
Immunocompromised§					
Yes	26 (17.8)	6 (14.6)	20 (19.0)	0.5	0.6
No	120 (82.2)	35 (85.4)	85 (81.0)		
Obesity					
Yes	45 (30.8)	13 (31.7)	32 (30.5)	0.5	0.7
No	92 (63.0)	27 (65.9)	65 (61.9)		
Unknown	9 (6.2)	1 (2.4)	8 (7.6)		
Diabetes mellitus					
Yes	33 (22.6)	13 (31.7)	20 (19.0)	0.06	0.3
No	112 (76.7)	27 (65.9)	85 (81.0)		
Unknown	1 (0.7)	1 (2.4)	0		
Hypertension					
Yes	50 (34.2)	14 (34.1)	36 (34.3)	0.3	0.1
No	95 (65.1)	26 (63.4)	69 (65.7)		
Unknown	1 (0.7)	1 (2.4)	0		
Chronic obstructive pulmonary disease or asthma					
Yes	22 (15.1)	8 (19.5)	14 (13.3)	0.2	0.1
No	123 (84.2)	32 (78.0)	91 (86.7)		
Unknown	1 (0.7)	1 (2.4)	0		
Median days after symptom onset (IQR)¶	9 (4–18)	5.5 (4–11)	12 (4–26)	<0.01	<0.01
Signs or symptoms at admission					
Fever					
Yes	76 (52.1)	22 (53.7)	54 (51.4)	1	0.6
No	66 (45.2)	18 (43.9)	48 (45.7)		
Unknown	4 (2.7)	1 (2.4)	3 (2.9)		
Cough					
Yes	93 (63.7)	28 (68.3)	65 (61.9)	0.8	0.5
No	49 (33.6)	12 (29.3)	37 (35.2)		
Unknown	4 (2.7)	1 (2.4)	3 (2.9)		
Headache					
Yes	29 (19.9)	8 (19.1)	21 (20.0)	0.7	0.7
No	110 (75.3)	30 (73.2)	80 (76.2)		
Unknown	7 (4.8)	3 (7.3)	4 (3.8)		
Fatigue					
Yes	40 (27.4)	12 (29.3)	28 (26.7)	0.6	0.8
No	99 (67.8)	26 (63.4)	73 (69.5)		
Unknown	7 (4.8)	3 (7.3)	4 (3.8)		
Gastrointestinal symptoms					
Yes	42 (28.8)	9 (22.0)	33 (31.4)	0.5	0.2
No	98 (67.1)	30 (73.2)	68 (64.8)		
Unknown	6 (4.1)	2 (4.9)	4 (3.8)		
Lymphopenia					
Yes	61 (41.8)	23 (56.1)	38 (36.2)	0.09	0.5
No	57 (39.0)	12 (29.3)	45 (42.9)		
Unknown	28 (19.2)	6 (14.6)	22 (21.0)		
Hospital admission					
Yes	81 (55.5)	24 (58.5)	57 (54.3)	0.6	0.5
No	65 (44.5)	17 (41.5)	48 (45.7)		
Intensive care unit admission					
Yes	30 (20.5)	9 (22.0)	21 (20.0)	0.8	0.8
No	116 (79.5)	32 (78.0)	84 (80.0)		
Mechanical ventilation during hospitalization					
Yes	14 (9.6)	5 (12.2)	9 (8.6)	0.5	0.6
No	132 (90.4)	36 (87.8)	96 (91.4)		
30-day all-cause death rate					
Yes	7 (4.8)	5 (12.2)	2 (1.9)	0.02	0.2
No	139 (95.2)	36 (87.8)	103 (98.1)		
Sample type					
Nasopharyngeal	134 (91.8)	37 (90.2)	97 (92.4)	0.8	0.1
Nasal	5 (3.4)	2 (4.9)	3 (2.9)		
Oropharyngeal	7 (4.8)	2 (4.9)	5 (4.8)		
Median nasopharyngeal cycle threshold value (IQR)#	29.9 (22.9–35.8)	20.8 (18.3–23.9)	33.4 (28.1–36.4)	<0.01	<0.01

*Values are no. (%) except as indicated. IQR, interquartile range; SARS-CoV-2, severe acute respiratory syndrome coronavirus 2. †p values determined by χ_2_ or Mann-Whitney U tests. ‡Adjusted for age, days after symptom onset, and cycle threshold value for standard SARS-CoV-2 PCR. §Immunocompromised status was defined as having an immuocompromising condition including hematologic or solid malignancy, history of stem cell or organ transplant, primary or secondary immunodeficiency, or drug-related immunocompromise. ¶19 values missing rrom patient medical records. #Cycle threshold value for standard SARS-CoV-2 PCR.

We detected the minus strand in 7 patients in the prospective cohort (Table 1). Two of these patients were nonimmunocompromised inpatients tested within >10 days after symptom onset, including 1 who had been asymptomatic for >48 hours; the C_t_ values for these samples were 39.0 and 38.6. We detected minus-strand SARS-CoV-2 RNA up to 30 days after symptom onset in an immunocompromised patient with persistent fever. For 2 patients in the prospective cohort, a negative result might have facilitated the approval of medical procedures despite prolonged positive results by standard rRT-PCR (Appendix).

## Conclusions

We described the performance of a 2-step strand-specific rRT-PCR for detection of SARS-CoV-2. The assay identified viral replication in patients with persistent positive results by standard rRT-PCR, possibly facilitating clinical decision-making. Other assays that assess intermediates of viral replication, such as subgenomic RNA, have emerged in the literature (*5*,*13*). Perera et al. demonstrated high correlation between levels of presumptive SARS-CoV-2 active replication intermediates and standard rRT-PCR C_t_ values (*13*). The standard SARS-CoV-2 rRT-PCR is appropriate for most routine clinical diagnostic applications. However, because this assay does not determine whether SARS-CoV-2 is actively replicating, it cannot infer infectiousness in samples with mid-level C_t_ values (i.e., C_t_ 25–35).

We detected minus-strand RNA up to 30 days after symptom onset, which is longer than the 14-day period previously reported for subgenomic RNA (*13*), and 8–15 day period for viral culture (*3*–*6*,*13*). We detected minus-strand RNA in 2 patients beyond the typical period recommended for isolation. Isolation strategies on the basis of time and symptoms are simple to apply, reduce the number of tests that need to be conducted, thus saving resources, and are probably effective at a population level (*14*). However, it can be challenging to determine the infectiousness of patients in certain clinical contexts, such as immunocompromised hosts with persistent viral shedding, on the basis of time and symptoms alone. Tools such as strand-specific RNA testing might be helpful in determining the infectiousness of these patients. Strand-specific testing might also help avoid delays in required procedures or treatments such as chemotherapy, which might be postponed because of SARS-CoV-2–positive PCR results.

This study has several strengths, including a large patient cohort and analytical validation. This strand-specific assay is useful because it can be adapted for routine clinical laboratory testing, does not require emergency use authorization, and reports C_t_ values and strand-specific RNA detection. The study was limited by its single-center design and combination of 2 patient cohorts chosen using different selection techniques. The assay lacks viral culture data and is hampered by longer turnaround time and complexity. In future studies, we will validate this assay against SARS-CoV-2 viral culture and within a household transmission study.

In summary, we described the test performance and clinical feasibility of a strand-specific rRT-PCR assay for SARS-CoV-2. Strand-specific rRT-PCR testing might be especially useful in patients with prolonged RNA shedding. It might also supplement existing strategies for estimating infectiousness on the basis of time and symptoms. Further work is required to correlate these findings with viral culture, compare different strand-specific RNA detection methods, and to assess clinical utility in large and longitudinal patient cohorts. These findings might improve understanding of the infectiousness of SARS-CoV-2, enabling optimization of infection control measures and resource use.

Appendix Additional information on strand-specific real-time reverse transcription PCR for detection of replicating SARS-CoV-2.

## Figures and Tables

**Table 2:** Clinical characteristics of 7 patients with SARS-CoV-2 minus-strand RNA, California, USA, 2020*

Patient ID	Age, y/sex	Immuno-compromised	Test order	Cycle threshold value for standard rRT-PCR specific to SARS-CoV-2	Days after symptom onset	Symptomatic improvement at time of strand-specific testing†	Fever within 24 h of strand-specific testing	Minus strand detected beyond CDC isolation recommendations‡
102	75/M	No	1	33.5	Unclear	Unclear§	No	Unclear
			2	19.7	Unclear	Unclear§	No	Unclear
111	58/M	Yes	1	NA¶	26	No	Yes	No
117	82/M	No	1	18.5	12	Unclear§	No	Yes
118	69/M	No	1	20.8	4	No	Yes	No
127	61/M	No	1	34.5	11	Yes	No	Yes
129	60/M	Yes	1	22.6	18	No	Yes	No
			2	20.2	30	No	Yes	No
141	2/F	Yes	1	18.1	NA#	NA#	NA#	NA#

*ID, identification; NA, not available; rRT-PCR, real-time reverse transcription PCR; SARS-CoV-2, severe acute respiratory syndrome coronavirus 2. †From symptom onset to time of testing. ‡According to recommendations from the Centers for Disease Control and Prevention (*14*). §Because of underlying condition. ¶Qualitative testing conducted at external reference laboratory. #Data not available because patient was treated at external medical facility.
