# Supplementary material for: Strand-Specific Reverse Transcription PCR for Detection of Replicating SARS-CoV-2
Source: Emerg Infect Dis. 2021 Feb;27(2):632–5. doi: 10.3201/eid2702.204168 (PMC7853532; doi:10.3201/eid2702.204168)
Supplement: Appendix — Additional information on strand-specific real-time reverse transcription PCR for detection of replicating SARS-CoV-2. [file 20-4168-Techapp-s1.pdf]

Article DOI: https://doi.org/10.3201/eid2702.204168

Strand-Specific Reverse Transcription PCR for Detection of Replicating SARS-CoV-2

Appendix

Routine SARS-CoV-2 Testing

All testing was performed at the Stanford Clinical Virology Laboratory (Stanford, CA, USA), a laboratory in northern California that serves 2 academic medical centers and affiliated clinics in the surrounding area. Standard clinical testing of severe acute respiratory syndrome coronavirus 2 (SARS-CoV-2) from respiratory samples was performed by the emergency use authorization for real-time reverse transcription PCR (rRT-PCR) specific to the SARS-CoV-2 envelope (*E*) gene, or with 1 of 2 commercial nucleic acid amplification test assays, the Panther Fusion SARS-CoV-2 (Hologic Inc., https://www.hologic.com), as previously described (*1*), or the Panther Aptima SARS-CoV-2 (Hologic Inc.). The range of cycle threshold values for the rRT-PCR assays was <20 cycles to 45 cycles, and samples with a cycle threshold (C_t) value >40 were repeated and considered positive if reproducible. Results from the Panther Aptima assay were not included for analysis in the C_t comparison of this study because only a few patients were tested with this assay and the lack of robust correlation between C_t values and relative light units.

Two-Step Strand-Specific PCR

RNA extraction was performed from 400µL of respiratory specimen using the EZ1 instrument (QIAGEN, https://www.qiagen.com). Strand-specific PCR testing consisted of 2 sets of reactions. In the first set of reactions, reverse transcription with strand-specific primers converted SARS-CoV-2 RNA to complementary DNA (cDNA). A reverse envelope (*E*) gene primer generated cDNA to the plus strand (rtR), comprising both genomic RNA and messenger RNA . In a separate reaction, a forward *E* gene reaction generated cDNA to the minus strand (rtF), comprising minus genomic and subgenomic RNA. A third reverse transcription reaction without an added primer was used as a control for each nucleic acid eluate (background (no

primer) reverse transcription reaction; rtX). In the second step, the cDNA was amplified by real-time PCR in 3 separate reactions using the Rotor-Gene Q instrument (QIAGEN) with the Stanford *E* gene assay.

The oligonucleotide sequences of the primers and probes are listed in Appendix Table 1. Each reaction contained 12µL of 2X reaction mix and 1.2µL of enzyme mix, both from the Luna Universal Probe One-Step RT-qPCR kit (New England Biolabs, Inc., https://www.neb.com), 2 µL of reverse transcription primers (rtF, rtR or rtX), and 10µL eluate. Reverse transcription (RT) was performed on the Veriti instrument (Thermo Fisher Scientific, https://www.thermofisher.com) for 15 minutes at 60°C, followed by RT inactivation for 10 minutes at 80°C. This corresponds to a time-based RT separation, rather than an enzyme-based RT separation. This modification was performed to maximize the eluate input volume into the PCR reaction. The reaction tubes were then removed from the thermal cycler, and 2µL of the Stanford EUA *E* gene assay primer/probe mix was added in the same tubes, as previously described (*2*). In the second step, the amplification step was resumed in the same reaction tube by real-time PCR (rRT-PCR) using the Rotor-Gene Q instrument (QIAGEN). Thermal cycling involved 2 minutes at 94°C, followed by 45 cycles of 15 seconds at 94°C, 40 seconds at 55°C, and 20 seconds at 68°C. Each run included a SARS-CoV-2-negative control (pooled nasopharyngeal specimens confirmed negative by standard SARS-CoV-2 rRT-PCR), and 2 positive controls (in vitro transcribed plus- and minus-strand RNA). The minus-strand target was reported as detected if the rtF reaction showed an exponential curve with a C_t <45 cycles, the rtX reaction was either not detected or detected ≥3 cycles later than the minus strand, and the rtR reaction was detected at a C_t less than that of the rtF reaction. Testing was performed as a laboratory-developed test for SARS-CoV-2 monitoring in a Clinical Laboratory Improvement Amendments–certified laboratory, and thus did not require emergency use authorization from the Food and Drug Administration.

Analytical Validation

To perform the analytical validation, minus- and plus-strand SARS-CoV-2 RNA was in vitro transcribed using the HiScribe T7 Quick Yield RNA Synthesis kit (New England Biolabs, Inc.) to be used as strand-specific control material. In vitro transcription was performed from T7-containing primers (Appendix Table 1), and one amplicon was generated for each direction. The

preparations were purified by DNAse treatment followed by lithium chloride precipitation. The control RNA material was quantitated using the *E* gene assay with single-stranded DNA as standard. The control RNA material was run in triplicate from 6.0 to 1.0 $\log_{10}$ copies/μL to evaluate linearity, and in triplicate at 10 and 100 copies/μL for 3 days to evaluate precision. The lower limit of detection (LLOD) was evaluated by testing 10 replicates each at 0.5, 1 and 2 copies/μL.

Research Cohort Selection (Retrospective Phase)

Patients whose initial strand-specific result was detected, and who had residual specimen leftover available from longitudinal samples, underwent repeat strand-specific testing. Repeat testing was not performed for individuals for whom initial strand-specific testing was negative. Specimens were stored at -80°C and thawed at the time of testing.

Clinical Cohort Selection (Prospective Phase)

Strand-specific rRT-PCR test results were reported in the electronic medical system. Results included the C_t value of the minus and plus strands, along with the respective interpretation as 'detected' or 'not detected' for each. Comments were autoappended to each strand-specific result to provide additional guidance on result interpretation. For positive results, the comment included that this was consistent with active virus replication. For negative results, the comment included a caution on the inability to rule out active virus replication on the basis of the strand-specific test result alone. Patients for whom testing had been ordered in error instead of routine RT-PCR for initial diagnosis were excluded. Test results were included for clinical decision-making on a case-by-case basis with support from the Stanford Infection and Prevention Control Program, and were not formally integrated into institutional guidelines at the time of this study. Chart review was performed as for the retrospective phase.

Statistical Analysis

The 95% LLOD was calculated by probit analysis using R version 4.0.2 (The R Project for Statistical Computing, https://www.r-project.org). Precision analysis was performed in Microsoft Excel (https://www.microsoft.com) as previously described (*15*). Clinical data statistical analysis was performed by $\chi 2$ test or Fisher's exact test when there were <5 datapoints per cell for categorical variables, or by the Mann-Whitney U test for continuous variables using

Stata version 15.1 (StataCorp LLC., https://www.stata.com). Multivariable logistic regression analysis was performed for the main clinical outcomes including age as a forced-in variable, and variables found to be significantly associated in bivariable analysis. Deming regression was performed to compare C_t results.

Results

Analytical Validation

The linear range extended from 1.0 to 6.0 log_{10} copies/μL for the minus and plus strands, with $R^2 = 0.99$ for both (Appendix Figure 1, panels A, B). Between-run and within-run precision showed coefficients of variation of <2% for both tested concentrations (Appendix Table 2). The 95% LLOD for the minus strand was calculated to be 0.5 RNA copies/μL (5 copies/reaction; 10/10 samples detected). Background signal was observed for all minus strand in vitro transcribed control material, with an average signal difference of 11.8 cycles between the minus strand and background. The background signal corresponded to valid amplification curves, which could not be distinguished from true positives. Additional DNAse treatment did not resolve this issue. Overall, these test performance characteristics were consistent with the performance seen with the Stanford *E* gene assay (B. Pinsky, unpub. data).

Clinical Results

The median C_t value from the standard nasopharyngeal rRT-PCR assay was 28.0 (interquartile range [IQR] 22.3–35.7) in the retrospective phase, and 33.4 (IQR 25.2–35.8) in the prospective phase, and the proportion of minus strand detection was significantly lower in the prospective phase (36.6% vs. 13.2%; $p<0.01$). A subset of 13 persons in the retrospective phase and 9 persons in the prospective phase underwent repeat strand-specific rRT-PCR testing, of whom a total of 4 tested positive for minus-strand RNA, up to 5 days after the first test. In the retrospective phase, background signal was detected in 8 (8.6%) of 93 samples, with a median C_t value of 39.3 (IQR 38.7–39.9) (Appendix Table 3). In contrast, no background signal was observed in the prospective clinical cohort testing (Appendix Table 4).

In 2 patients in the prospective cohort, a negative result for minus-strand RNA facilitated the approval to proceed with procedures in the context of persistently positive SARS-CoV-2 rRT-PCR results. The first case included approval to proceed with surgery for a woman 18 years

of age for tumor investigation and management despite positive SARS-CoV-2 rRT-PCR results after >10 days of home isolation for coronavirus disease. The second case included facilitating approval to list an immunocompromised woman 56 years of age for organ transplant after >50 days of rRT-PCR positivity. Both of these presented with positive Aptima results, 1,135 and 1,027 relative light units, respectively.

References

1. Hogan CA, Sahoo MK, Huang C, Garamani N, Stevens B, Zehnder J, et al. Comparison of the Panther Fusion and a laboratory-developed test targeting the envelope gene for detection of SARS-CoV-2. J Clin Virol. 2020;127:104383. PubMed https://doi.org/10.1016/j.jcv.2020.104383

2. U.S. Food and Drug Administration. Stanford Health Care Clinical Virology Laboratory SARS-CoV-2 test EUA Summary. 2020 Nov 24 [cited 2020 Dec 2]. https://www.fda.gov/media/136818/download

Appendix Table 1. Strand-specific PCR primer and probe specifications in study on strand-specific reverse transcription PCR specific for detection of replicating severe acute respiratory syndrome coronavirus 2, California, USA, 2020*

Name	Concentration, nmol/L	Sequence (5’→3’)
Reverse transcription primers		
nCoVsg_E.rtF	400	CGAACTTATGTACTCATTCGTTTCGG
nCoVsg_E.rtR	400	AGAAGGTTTTACAAGACTCACGTT
E gene real-time PCR primers		
SARS-CoV-2_E_Fwd	400	ACAGGTACGTTAATAGTTAATAGCGT
SARS-CoV-2_E_Rev	400	ATATTGCAGCAGTACGCACACA
RNase P Forward	200	AGATTTGGACCTGCGAGCG
RNase P Reverse	200	GAGCGGCTGTCTCCACAAGT
E gene real-time PCR probes		
SARS-CoV-2_E_Prb-FAM	200	ACACTAGCCATCCTTACTGCGCTTCG
RNase P Cf-560	50	TTCTGACCTGAAGGCTCTGCGCG
In vitro transcription primers		
nCoVsg_E.FO.T7	400	TAATACGACTCACTATAGGG CTTTGTAAGCACAAGCTGATGAGT
nCoVsg_E.RO	400	CCAGAAGATCAGGAACTCTAGAAGA
nCoVsg_E.FO	400	CTTTGTAAGCACAAGCTGATGAGT
nCoVsg_E.Ro.T7	400	TAATACGACTCACTATAGGG CCAGAAGATCAGGAACTCTAGAAGA

**E* gene, envelope gene; SARS-CoV-2, severe acute respiratory syndrome coronavirus 2; Rtf, forward envelope gene reaction; RtR, reverse envelope gene reaction.

Appendix Table 2. Run results of study on strand-specific real-time reverse transcription PCR specific for detection of replicating severe acute respiratory syndrome coronavirus 2, California, USA, 2020*

RNA	Mean cycle threshold value	Within run†		Between runs†		Total	
		SD	CV, %	SD	CV, %	SD	CV, %
Minus strand, cp/µL							
100	30.7	0.15	0.49	0.52	1.70	0.54	1.75
10	34.1	0.30	0.87	0.53	1.54	0.58	1.70
Plus strand, cp/µL							
100	29.3	0.33	1.12	0.40	1.36	0.48	1.64
10	32.8	0.22	0.68	0.27	0.81	0.32	0.98

*cp/µL, copies per microliter; CV: coefficient of variation.
†The control RNA material was run in triplicate at 10 copies/µL and 100 copies/µL each day for 3 days to evaluate precision. Each run included a negative control (0cp/mL); the results of the negative control were not detected.

Appendix Table 3. Full results of the strand-specific real-time reverse transcription PCR for retrospective clinical samples from patients with severe acute respiratory syndrome coronavirus 2, California, USA, 2020*

Patient ID	Sample type	Cycle threshold values			Cycle threshold by standard PCR†
		Background	Minus strand	Plus strand	
1	NP	ndet	32.6	16.6	16.4
2	NP	ndet	37.4	23.5	26.4
3	NP	ndet	30.4	15.7	18.5
4	NP	ndet	ndet	ndet	37.5
5	NP	ndet	ndet	28.4	29.9
6	NP	ndet	37.8	24.9	25.8
7	NP	ndet	36.0	21.3	20.7
8	NP	ndet	ndet	31.7	32.5
9	NP	ndet	34.0	19.4	20.3
10	NP	ndet	39.2	22.2	21.3
11	NP	39.0	ndet	35.2	35.9
12	NP	ndet	34.9	18.9	15.6
13	NP	ndet	32.4	17.0	19.3
14	NP	ndet	34.1	17.5	15.3
15	NP	ndet	ndet	32.3	25.2
16	NP	ndet	ndet	30.8	35.9
17	NP	ndet	32.6	16.6	15.0
18	NP	ndet	ndet	30.5	33.2
19	NP	ndet	39.1	25.9	24.8
20	NP	39.7	ndet	26.0	23.2
21	NP	ndet	35.9	26.6	27.1
22	NP	ndet	ndet	28.1	27.0
23	NP	ndet	30.8	20.2	18.9
24	NP	ndet	ndet	39.3	38.6
25	NP	ndet	ndet	29.3	29.8
26	NP	ndet	ndet	ndet	34.5
27	NP	ndet	ndet	ndet	40.5
28	NP	ndet	ndet	34.7	32.6
29	NP	ndet	34.7	20.8	23.5
30	NP	ndet	ndet	27.1	27.3
31	NP	ndet	ndet	28.7	30.1
32	NP	ndet	ndet	34.2	35.4
33	NP	37.5	27.4	14.3	16.4
34	NP	ndet	ndet	34.5	36.2
35	NP	ndet	ndet	38.7	39.5
36	NP	ndet	ndet	32.6	35.7
37	NP	ndet	ndet	32.9	36.8
38	NP	ndet	ndet	33.0	36.7
39	NP	ndet	ndet	36.3	41.8
40	NP	ndet	ndet	35.5	39.5
41	NP	ndet	ndet	27.4	30.0
42	NP	ndet	33.2	18.5	19.0
43	NP	ndet	ndet	31.9	31.6
44	NP	ndet	31.2	20.3	23.3
45	NP	ndet	38.6	24.4	24
46	NP	ndet	ndet	22.2	23.9
47	NP	ndet	ndet	36.7	36.5

48	NP	38.8	ndet	28.5	29.2
49	NP	ndet	ndet	35.5	33.4
50	NP	ndet	ndet	32.1	35.7
51	NP	ndet	ndet	32.2	32.2
52	NP	38.5	31.9	15.9	16.3
53	NP	ndet	ndet	35.5	33.2
54	NP	41.1	ndet	31.6	32.3
55	NP	ndet	37.5	22.1	23.9
56	NP	ndet	ndet	34.5	34.1
57	NP	39.9	32.2	16.8	13.5
58	NP	ndet	ndet	28.9	25.1
59	OP	ndet	ndet	28.4	24.4
60	NP	ndet	ndet	28.4	26.5
61	NP	ndet	32.2	19.1	16.7
62	NP	ndet	36.7	23.6	24.9
63	NP	ndet	ndet	29.1	28.0
64	NP	ndet	ndet	27.1	24.9
65	Nasal	ndet	39.3	24.0	19.3
66	OP	ndet	ndet	29.5	23.2
67	OP	ndet	38.8	22.5	18.9
68	NP	ndet	36.4	21.2	20.8
69	NP	ndet	39.9	21.5	21.2
70	NP	ndet	37.4	23.3	22.3
71	NP	ndet	ndet	28.7	28
72	Nasal	ndet	39.8	28.6	24.1
73	NP	ndet	ndet	32.6	33.1
74	NP	ndet	ndet	36.3	36.9
75	NP	ndet	ndet	28.6	29.4
76	NP	ndet	39.3	24.8	22.2
77	NP	ndet	ndet	36.5	36.4
78	NP	ndet	ndet	38.0	42.7
79	NP	ndet	ndet	35.3	36.4
80	NP	ndet	ndet	ndet	37.1
81	NP	ndet	36.1	18.0	18.3
82	OP	ndet	39.4	26.3	21.8
83	NP	40.1	33.3	14.4	16.8
84	NP	ndet	ndet	29.6	30.5
85	NP	ndet	ndet	24.6	25.0
86	OP	ndet	ndet	34.3	27.7
87	NP	ndet	ndet	36.7	35.5
88	NP	ndet	ndet	ndet	35.9
89	NP	ndet	ndet	ndet	37.3
90	NP	ndet	ndet	ndet	38.5
91	OP	ndet	ndet	ndet	35.9
92	NP	ndet	ndet	34.8	30.9
93	OP	ndet	ndet	ndet	37.5

*ndet, not detected; NP, nasopharyngeal; OP, oropharyngeal.

Appendix Table 4. Full results of the strand-specific real-time reverse transcription PCR assay for prospective clinical samples from patients with severe acute respiratory syndrome coronavirus 2, California, USA, 2020*

		Cycle threshold values			
Patient ID	Sample type	Background	Minus strand	Plus strand	Cycle threshold by standard PCR or relative light units by transcription-mediated amplification†
94	NP	ndet	ndet	39.6	ndet
95	NP	ndet	ndet	35.9	34.2
96	NP	ndet	ndet	34.6	1,135†
97	NP	ndet	ndet	ndet	40.1
98	NP	ndet	ndet	ndet	584†
99	NP	ndet	ndet	31.1	NA
100	NP	ndet	ndet	31.2	31.0
101	NP	ndet	ndet	ndet	NA
102	NP	ndet	28.7	16.1	NA
103	NP	ndet	ndet	38.7	37.3
104	NP	ndet	ndet	ndet	NA
105	NP	ndet	ndet	32.2	1,144†
106	NP	ndet	ndet	38.9	35.2
107	NP	ndet	ndet	35.2	1,057†
108	NP	ndet	ndet	34.9	21.4
109	NP	ndet	ndet	34.5	34.4
110	Nasal	ndet	ndet	36.5	NA
111	NP	ndet	27.9	13.0	NA
112	NP	ndet	ndet	37.6	36.6
113	Nasal	ndet	ndet	ndet	38.0
114	NP	ndet	ndet	38.4	35.8
115	NP	ndet	ndet	25.9	27.2
116	NP	ndet	ndet	34.8	33.6
117	NP	ndet	39.0	23.7	18.5
118	NP	ndet	33.2	19.2	20.8
119	NP	ndet	ndet	ndet	1,140†
120	NP	ndet	ndet	26.4	25.2
121	NP	ndet	ndet	ndet	1,027†
122	NP	ndet	ndet	34.7	35.1
123	NP	ndet	ndet	35.3	34.7
124	NP	ndet	ndet	38.5	783†
125	NP	ndet	ndet	34.0	776†
126	NP	ndet	ndet	30.9	33.4
127	NP	ndet	38.6	31.9	34.5
128	NP	ndet	ndet	37.3	NA
129	NP	ndet	34.3	21.1	22.6
130	NP	ndet	ndet	34.7	32.1
131	Nasal	ndet	ndet	27.0	29.3
132	NP	ndet	ndet	ndet	1,282†
133	NP	ndet	ndet	ndet	37.6
134	NP	ndet	ndet	26.2	28
135	NP	ndet	ndet	ndet	18.6
136	NP	ndet	ndet	37.5	35.8
137	NP	ndet	ndet	32.77	33.1
138	NP	ndet	ndet	ndet	36.9
139	NP	ndet	ndet	35.48	1,084†
140	NP	ndet	ndet	34.94	36.8
141	NP	ndet	27.61	16.09	18.1
142	NP	ndet	ndet	35.22	36.1
143	NP	ndet	ndet	ndet	38.4
144	NP	ndet	ndet	ndet	957†
145	NP	ndet	ndet	34.04	30.94
146	NP	ndet	ndet	36.55	NA

*NA, not available; ndet, not detected; NP, nasopharyngeal.
†Samples were tested by real-time reverse transcription PCR or by transcription-mediated assay at the Stanford Clinical Virology Laboratory, Stanford, CA, USA. Crosses (†) indicate samples tested on the transcription-mediated assay, for which the results are expressed as relative light units. NA values indicate tests that were performed at external laboratories.

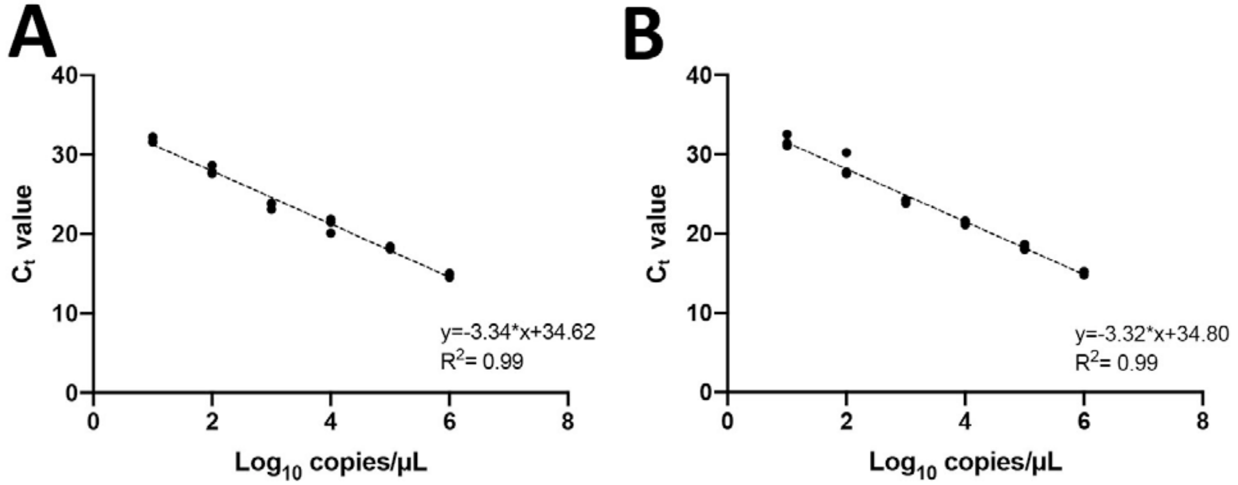

Appendix Figure 1. Linearity of Ct values obtained by strand-specific real-time reverse transcription PCR for the SARS-CoV-2 (A; y = –3.34x+34.62; R2 = 0.99) minus strand and (B; y = –3.32x+34.80; R2 = 0.99) plus strand, California, USA, 2020. Ct, cycle threshold; SARS-CoV-2, severe acute respiratory syndrome coronavirus 2.

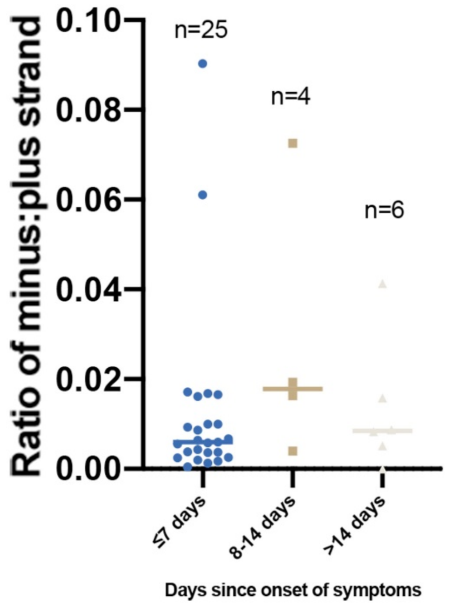

Appendix Figure 2. Ratio of minus:plus strand viral load in samples from patients with severe acute respiratory syndrome coronavirus 2 infection, California, USA, 2020. The dataset included 38 samples for which time to symptom onset data were available. Three outliers were excluded from this figure for improved visualization: 1 sample collected ≤7 days after symptom onset (y = 3.44), and 2 samples collected 8–14 days after symptoms onset (y = 1.18 and y = 0.21).
